# In Situ Study on Fracture Behavior of Z-Pinned Carbon Fiber-Reinforced Aluminum Matrix Composite via Scanning Electron Microscope (SEM)

**DOI:** 10.3390/ma12121941

**Published:** 2019-06-17

**Authors:** Yunhe Zhang, Sian Wang, Xiwang Zhao, Fanming Wang, Gaohui Wu

**Affiliations:** 1 College of Mechanical and Electrical Engineering, Northeast Forestry University, P.O. Box 310, Hexing Road No.26, Harbin 150040, China; daowsa@nefu.edu.cn (S.W.); zhaoxiwang1216@126.com (X.Z.); 18512490598@163.com (F.W.); 2 School of Materials Science and Engineering, Harbin Institute of Technology, Harbin 150080, China; wugh@hit.edu.cn

**Keywords:** electron microscopy (in situ SEM), delamination, metal matrix composites (MMCs), z-pinning

## Abstract

Inside a scanning electron microscope (SEM) chamber, we performed an in situ interlaminar shear test on a z-pinned carbon fiber-reinforced aluminum matrix composite (Cf/Al) fabricated by the pressure the infiltration method to understand its failure mechanism. Experiments show that introducing a stainless-steel z-pin increases the interlaminar shear strength of Cf/Al composite by 148%. The increase in interlaminar shear strength is attributed to the high strength of the stainless-steel z-pin and the strong bonding between the z-pin and the matrix. When the z-pin/matrix interface failed, the z-pin can still experience large shear deformation, thereby enhancing delamination resistance. The failure mechanism of composite includes interfacial debonding, aluminum plough, z-pin shear deformation, frictional sliding, and fracture. These results in this study will help us understand the interlaminar strengthening mechanism of z-pins in the delamination of metal matrix composites.

## 1. Introduction

Carbon fiber-reinforced aluminum matrix composites (Cf/Al) have drawn great attention in automotive and aerospace applications due to their high specific strength, high specific modulus, high thermal conductivity, and good fatigue properties [1,2,3]. However, a major concern with the application of Cf/Al laminated composites is the delamination that results from through-thickness stresses or impact loads, which lead to the reduction of the in-plane mechanical properties and even structural instability of the component [4,5]. Z-pinning is an effective technique that enhances delamination resistance and interlaminar strength of laminates by inserting metallic or fibrous rods (i.e., z-pins) in the through-thickness direction of laminated composites during their fabrication process [6]. A great number of studies have shown that delamination fracture toughness, interlaminar shear strength, and damage tolerance of composite laminates can increase significantly with z-pins [7,8,9].

Zhang et al. studied the influence of z-pinning on the interlaminar mechanical properties of Cf/Al composites fabricated by the pressure infiltration method [10]. They found that the stainless-steel z-pin creates a strong bond with the composite due to an interface reaction between the z-pin and the aluminum matrix, improving the interlaminar shear strength of the composite by at least 70%. Although their study determined the capacity of the z-pin for improving delamination resistance, it did not give a clear understanding of the failure mechanism in z-pinned Cf/Al composites. Thus, before using z-pinned Cf/Al composites in aerospace structures, it is necessary to understand their mechanical performance and strengthening mechanism under interlaminar shear load.

Researchers have performed several experimental studies to investigate the interlaminar strengthening mechanisms of z-pinned carbon fiber-reinforced polymer matrix composites, such as double cantilever beam (DCB) fracture tests, end notch flexure (ENF) fracture tests, z-pin pull-out tests, and interlaminar shear tests [4,11,12]. They have effectively exploited the in situ scanning electron microscope (SEM) technique to observe the fracture process of composites, since it can help us observe the fracture behavior of other types of composite structures in real time [13,14,15]. Nevertheless, the SEM technique has not hitherto been used to investigate the interlaminar fracture process of z-pinned composite structures.

This study builds on the recent work by Zhang et al. [10], which investigated the effect of z-pinning on microstructure and interlaminar shear strength of Cf/Al composites. In the current study, unpinned and z-pinned Cf/Al composites were fabricated by pressure infiltration method and double-notched interlaminar shear tests were applied to the unpinned and z-pinned Cf/Al specimens. Using the in situ SEM technique, the different interlaminar fracture behaviors of the z-pinned Cf/Al specimen were observed, such as the initiation and growth of crack, interfacial debonding, and the deformation of the z-pin and the matrix. Finally, the impact of z-pinning on fracture processes of Cf/Al composites were discussed.

## 2. Experimental

We used M40 carbon fibers (purchased from Toray Industries Inc., Tokyo, Japan) and AISI 321 stainless steel (purchased from Shanghai Baosteel Group Corporation, Shanghai, China) to reinforce 5A06 Al matrix (purchased from Northern Light Alloy Company Ltd., Harbin, China). Table 1 outlines the mechanical properties of 5A06 Al, M40 carbon fiber, and AISI 321. Table 2 and Table 3 list the chemical compositions of AISI 321 and 5A06 Al, respectively. 

We used the pressure infiltration method to fabricate the z-pinned and unpinned Cf/Al composites. The carbon fiber bundles were unidirectionally winded by a CNC winding machine to obtain the preform of carbon fibers, and an AISI 321 z-pin with a diameter of 0.6 mm was inserted into the preform by an ultrasonic tool. The preform of carbon fibers with the z-pin was preheated at 500 ± 10 °C. The melted 5A06 Al alloy was infiltrated into the preforms under pressure. During the infiltration process, a pressure of 0.5 MPa was applied and maintained for 2 h, and then the z-pinned Cf/Al composites were solidified in air. An unpinned Cf/Al composite was fabricated in the same route as the reference material. The composites had a carbon fiber volume fraction of about 55%.

**Figure 1:**
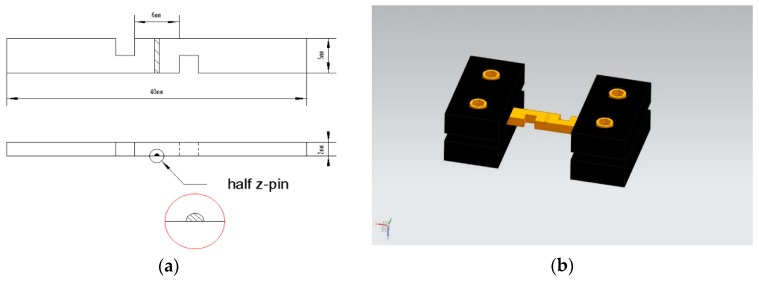
(**a**) The dimensions of the specimen for in situ observation. (**b**) The three-dimensional model of the specimen for in situ observation with the fixture.

The double-notched interlaminar shear specimens were machined by electric discharge using an electric discharge machining method. The distance between the two notches was 6 mm, and the depth of each notch was half of the specimen thickness (i.e., 2.5 mm) to make sure that the plane between the ends of the two notches was subjected to pure interlaminar shear loads. The single z-pin used to reinforce the specimen was equidistant from the two notches. Since the SEM technique can only observe the surface of the specimens, we cannot directly capture the deformation of the z-pin, crack initiation, and propagation of interfacial zone during in situ SEM observations. Hence, to obtain this information, we cut the specimen for in situ observation by an electric discharge along the central axis of the stainless-steel z-pin and half of the z-pin was still bonded with the specimen. It is worth noting that the cutting process does not induce the initial delamination. Figure 1a shows the dimensions of the specimen for in situ observation with the location of half z-pin. Figure 1b shows the three-dimensional model of the specimen for in situ observation with the fixture.

We conducted an in situ double notch interlaminar shear test in an S-4700 SEM equipped with a tensile stage (SEM, Royal Dutch Philips Electronics Ltd., Amsterdam, The Netherlands), which can be used to apply interlaminar shear loads. The test was performed using a cross-head speed of 0.5 mm/min at room temperature according to the ASTM D3846-2008. During the tests, we gathered the computer-generated load-displacement curves. The loading can be paused at any time to allow in situ observation. We carried out in situ SEM observations mainly on the notch and the interfacial zone between the z-pin and the aluminum matrix where the initial damage can be expected due to stress concentration.

## 3. Results and Discussion

**Figure 2:**
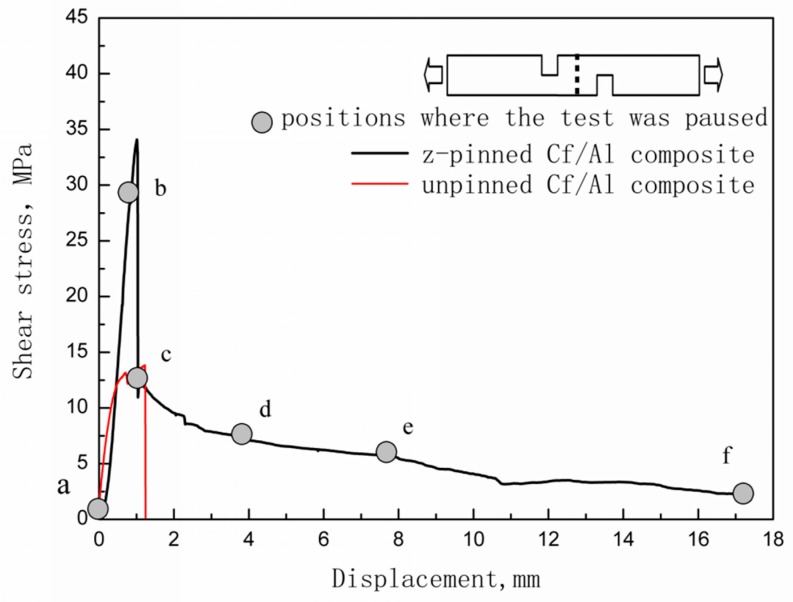
Typical tensile shear curves of carbon fiber-reinforced aluminum matrix composites (Cf/Al) with and without a z-pin.

Figure 2 shows the typical tensile shear curve for Cf/Al composites reinforced with half of the stainless-steel z-pin with a diameter of 0.6 mm. It also shows the representative curve for the unpinned Cf/Al composites for comparison. Table 4 summarizes the interlaminar shear strengths for the z-pinned specimen with a half z-pin for in situ observation, the z-pinned specimen with the entire z-pin, and the control specimen. The resulting numbers indicate that the interlaminar shear strength of the z-pinned Cf/Al composite is higher than the Cf/Al composite without a z-pin reinforcement by 148%. While the measured interlaminar shear strength of Cf/Al composites reinforced with half of the stainless-steel z-pin is slightly lower than that of Cf/Al composites reinforced with the entire stainless-steel z-pin in the previous work, the tensile shear curve for Cf/Al composites reinforced with half of the stainless-steel z-pin is consistent with that of Cf/Al composites reinforced with the entire stainless-steel z-pin with a diameter of 0.6 mm [10]. This shows that the cutting of the specimen and the z-pin does not change the fracture behavior of z-pinned Cf/Al composites. Hence, direct observation of a half z-pin-reinforced specimen via in situ tensile tests is an effective method for observing the fracture process of z-pinned Cf/Al composites.

**Figure 3:**
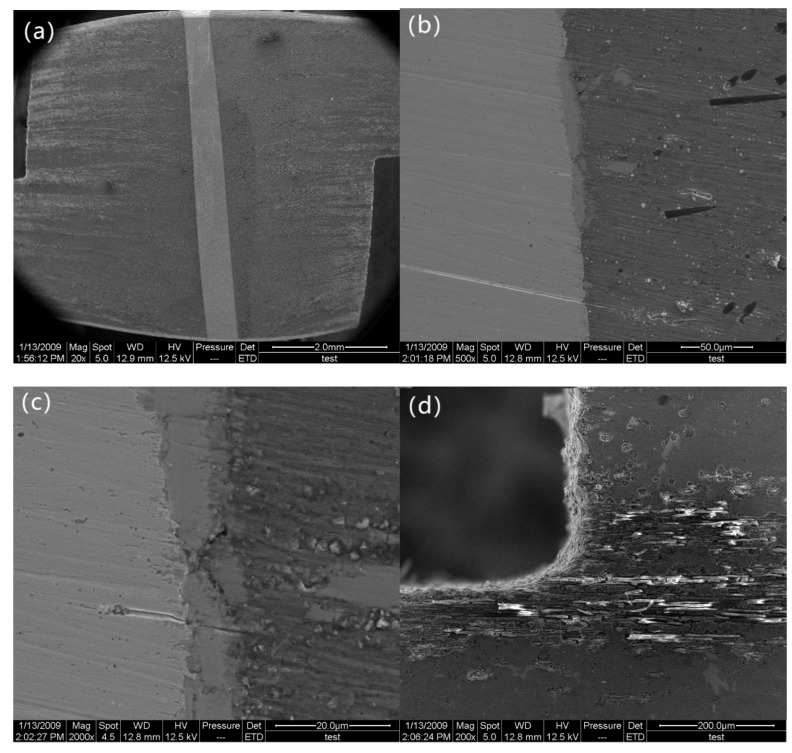
Morphology of the specimen prior to test: (**a**) the z-pin, (**b**) the z-pin/matrix interface in low magnification, (**c**) the z-pin/matrix interface in high magnification, and (**d**) the notch.

Figure 3 shows the SEM images of the specimen prior to the double-notch tensile test corresponding to point A in the curve. As shown in Figure 3a, the z-pin is almost perpendicular to the loading direction and carbon fiber (it is impossible to obtain a specimen with a perfectly perpendicular z-pin). As Figure 3b,c indicate, the z-pinned Cf/Al specimen shows the extent of the interface reaction layer at z-pin/matrix interface, which is about 10 μm in thickness. The main component of the interface reaction layer was confirmed to be FeAl_3_ by energy disperse spectroscopy in previous research [10]. This suggests that the z-pins hold a strong bond to the matrix. Moreover, no cracks are found in the matrix and the z-pin/matrix interface layer, as shown in Figure 3d.

**Figure 4:**
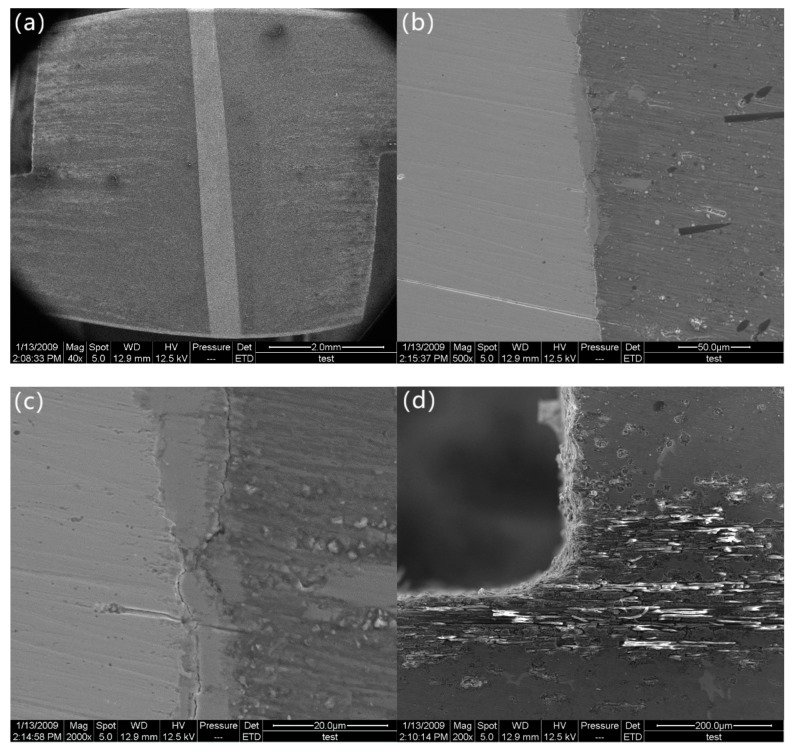
Morphology of specimen in later elastic deformation stage: (**a**) the z-pin, (**b**) micro-cracks in z-pin/matrix interface in low magnification, (**c**) micro-cracks in z-pin/matrix interface in high magnification, and (**d**) the notch.

Initially, the z-pinned composite exhibits an initial elastic region until it reaches maximum load. As the stress builds up to 28.3 MPa (marked as “b” in Figure 2), a clear deformation of the z-pin is not observed, which suggests that the z-pin only experiences elastic deformation (Figure 4a). Micro-cracks are observed in the z-pin/matrix interface at the mid-plane and they are located in the interfacial layer bonded with the stainless-steel z-pin (Figure 4b,c). However, the interface is not cracked and a good interfacial bonding also remains. As shown in Figure 4d, the specimen is not damaged, which reveals that delamination did not initiate. Therefore, it can be concluded that in spite of the presence of micro-cracks, the interlaminar shear stress is effectively transferred from the matrix to the z-pin through the z-pin/matrix interface. The stainless steel can carry the applied load due to its high strength and stiffness, resulting in the improved interlaminar shear strength. Hence, the interlaminar shear strength of the Cf/Al composite is enhanced owing to the high shear strength and stiffness of the introduced stainless-steel z-pin and the strong interfacial bonding between the z-pin and the matrix.

**Figure 5:**
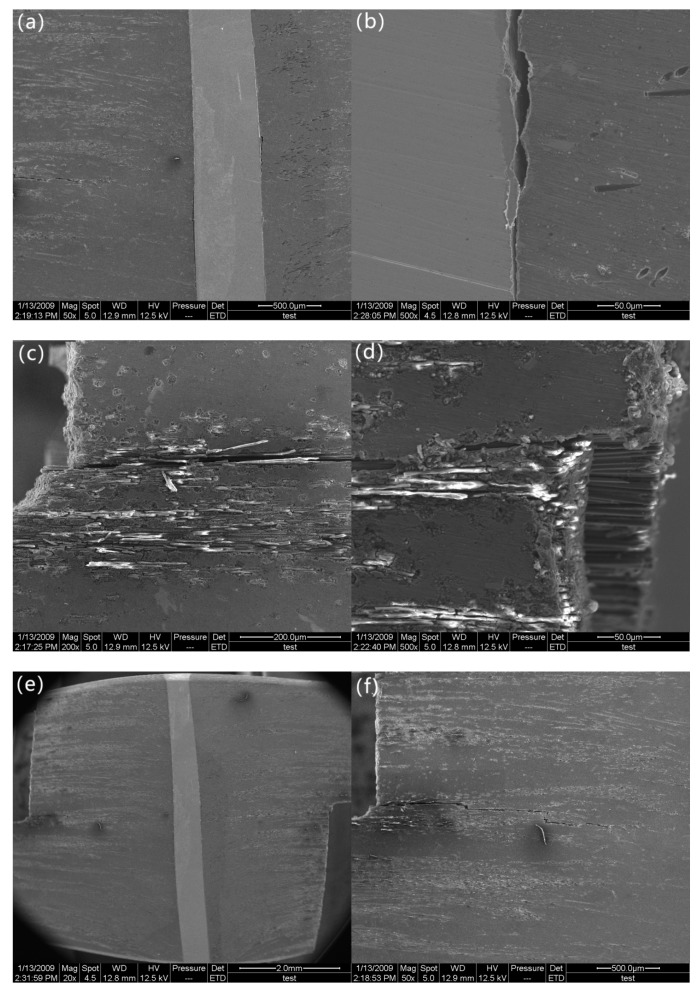
Morphology of the specimen after delamination initiates: (**a**) partial debonding at low magnification, (**b**) partial debonding at high magnification, (**c**) the notch at low magnification, (**d**) the notch at high magnification, (**e**) delamination crack growth at low magnification, and (**f**) delamination crack growth at high magnification.

After reaching the maximum load (34.1 MPa), the stress rapidly decreases until it reaches 11.5 MPa. As the stress decreases to 11.5 MPa (marked as “c” in Figure 2) micro-cracks propagate along the pin/matrix interface, which result in partial debonding at the midplane (Figure 5a,b). As a result, a partial interface cannot be effectively transferred from the matrix to the stainless-steel z-pin. At this point, the carbon fiber/matrix interface becomes the carrier that bears the applied interlaminar shear load. However, the carbon fiber/matrix interface near the notches that are in the stress concentration area cannot sustain the applied load due to low carbon fiber/matrix interfacial strength, which leads to debonding initiation of the carbon fiber/aluminum matrix. For this reason, delamination initiates at the plane between the ends of the two notches (Figure 5c,d) but still does not propagate throughout the entire plane (Figure 5e,f). The shear stress exhibits a load drop in the tensile shear curve as a result of delamination initiation. As shown in Figure 5e, the z-pin experiences permanent S-shaped shear deformation due to shear sliding displacement of the composite laminate, which in turn resists further delamination of the composite [16]. With the gradual rotation of the deformed z-pin axis, the partial interlaminar shear stress applied to the z-pin is decomposed into tensile stress along the z-pin direction. The z-pin loading mode transitions from shear to a combination of shear and tension. This allows the deformed z-pin to withstand relatively high stresses due to its high tensile strength. In addition, the z-pin/matrix interface near the z-pin ends still creates strong bonds, which suggests that the interface still retains a certain capacity to transfer the load. Since the z-pin/matrix interface is not debonded and the z-pin is not damaged, the specimen still has a relatively high carrying capacity (11.5 MPa), which is close to the maximum interlaminar shear stress value of the unpinned Cf/Al composite during the entire test (13.8 MPa). Hence, it can be also summarized that the strong bonding between the z-pin and the matrix plays a significant role in determining the interlaminar shear strength.

**Figure 6:**
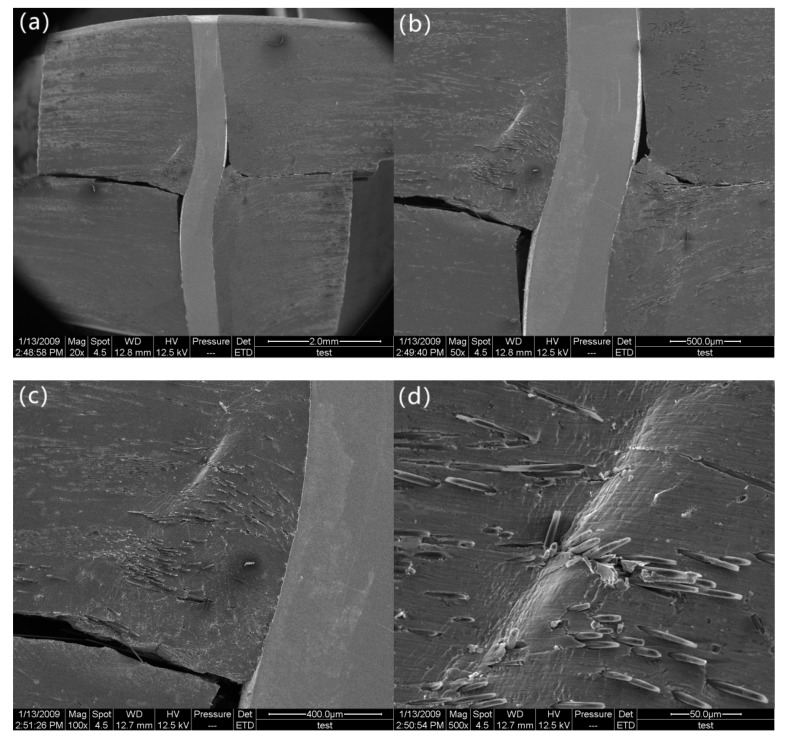
Morphology of the specimen when the matrix is deformed: (**a**) deformed z-pin at low magnification, (**b**) deformed z-pin at high magnification, (**c**) deformed matrix at low magnification, and (**d**) deformed matrix at high magnification.

**Figure 7:**
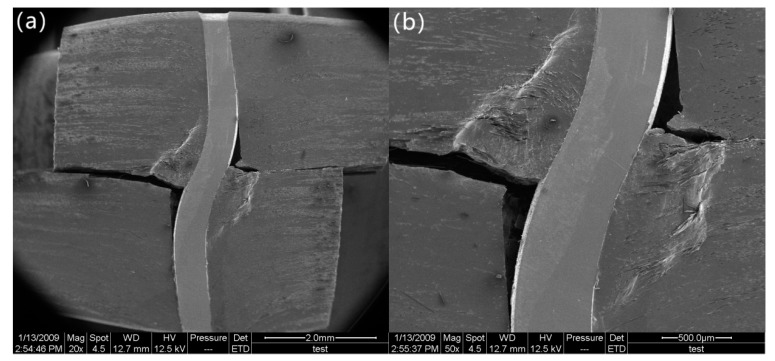
Morphology of the specimen when z-pin/matrix interface is fully debonded: (**a**) the z-pin sliding and pulling out, (**b**) the ploughed matrix.

After the load drops to 11.5 MPa, it begins to decrease slowly. At a stress of 7.6 MPa (marked as “d” in Figure 2), the delamination crack develops from the notches to the z-pin, where delamination propagation is suppressed due to the presence of the z-pin (Figure 6a,b). In addition, the z-pin is severely deformed and pressed laterally into the specimen matrix due to further interlaminar shear displacement of the composite. For this reason, the composite matrix adjacent to the stainless-steel z-pin is severely deformed (Figure 6c,d). At a stress of 6 MPa (marked as “e” in Figure 2), the segment of the z-pin below the delamination surface is pulled slightly outward from the aluminum matrix under interlaminar shear loading. The other segment of the z-pin above the delamination surface apparently slides along the interface due to the pull-out of the z-pin’s lower part, as shown in Figure 7a. All these observations indicate that the z-pin/matrix interface has completely failed. Nevertheless, it is hard to know the precise moment at which the failure occurred. This is because even if the interface is completely debonded, the z-pin and the matrix will still be close together due to the compression force applied by the matrix before the z-pin is pulled out. The friction between the sliding z-pin and the surrounding matrix during the pull-out process contributes to the interlaminar strengthening of z-pinned composites [17]. In addition, as seen in Figure 7b, the aluminum matrix close to the delamination surface is significantly ploughed out due to the further lateral deflection of the z-pin, which significantly enhances the interface friction between the z-pin and aluminum matrix and thereby improves the delamination resistance at this stage. The strengthening process that involves lateral compression deformation and the plough of the matrix induced by the z-pin is called the snubbing effect [18,19]. Hence, the interlaminar shear strength of the z-pinned Cf/Al composite during this stage is attributed to the snubbing effect and lateral deflection of the z-pin.

**Figure 8:**
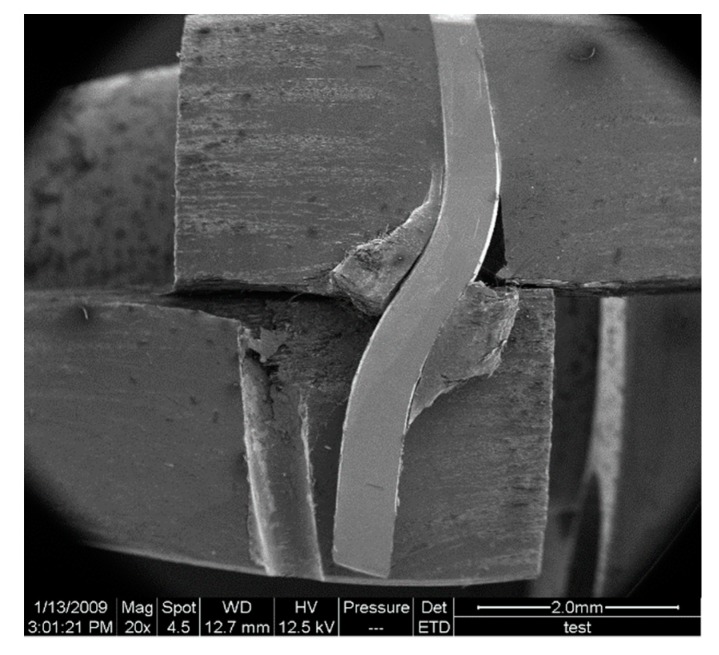
Morphology of the failed specimen for in situ observation.

Finally, at a stress level of 2.3 MPa, the z-pin fails by the outward pull-out, resulting in the final failure of the z-pinned Cf/Al specimen, as shown in Figure 8. It must be noted that if the specimen had not been cut along the central axis of the stainless-steel z-pin and the entire z-pin had still been surrounded by the aluminum matrix, the outward pull-out of the z-pin would have been restrained by the surrounding aluminum matrix. Therefore, the z-pin would have been intended to rupture with minimal pull-out rather than the outward pull-out reported in this study [10].

Previous studies on the mechanical properties of Cf/Al composites have revealed that the interlaminar shear strength of the unpinned Cf/Al composites depends on the interface strength between carbon fiber and the aluminum matrix [20]. Since the carbon fiber/matrix interface strength is poor, low interlaminar shear load causes debonding in the carbon fiber/aluminum interface, resulting in delamination initiation. With the increase in displacement, the delamination gradually develops along the debonded carbon fiber/aluminum interface, leading to the ultimate failure of the Cf/Al specimen. Thus, an unpinned Cf/Al composite shows low interlaminar shear strength.

**Figure 9:**
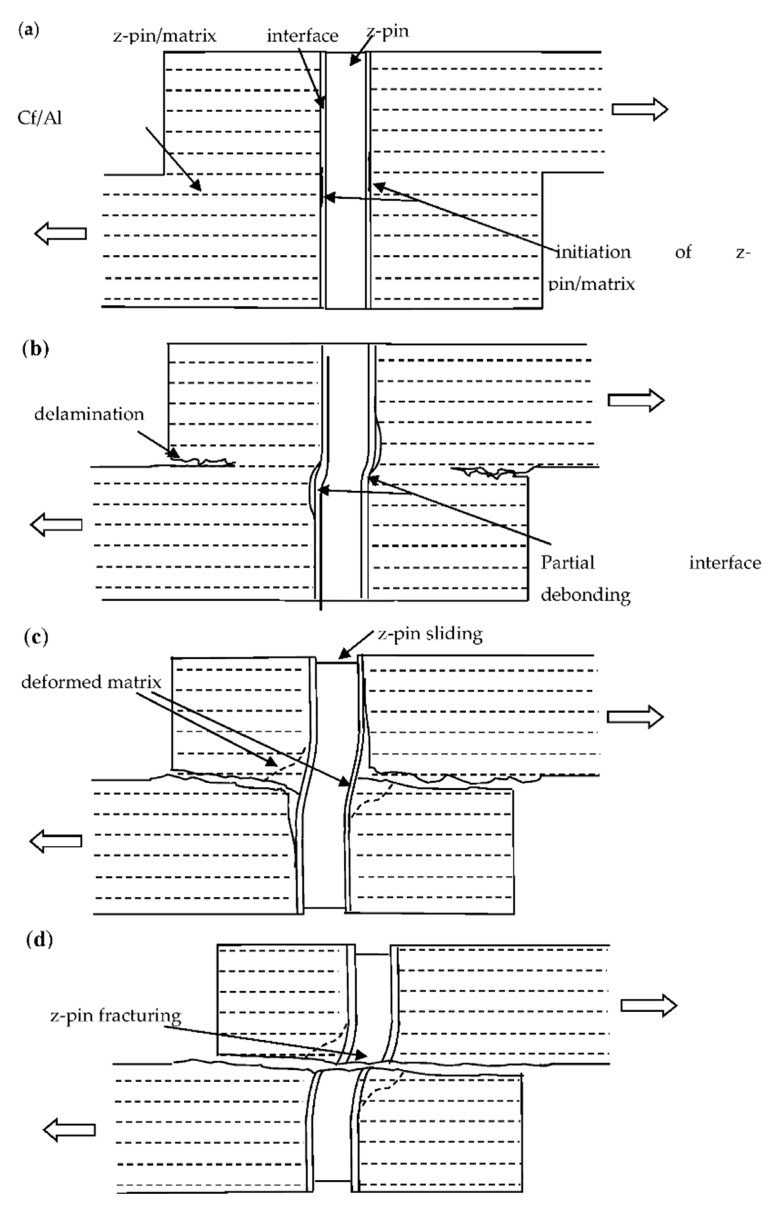
The schematic of the failure process of the z-pinned composites: (**a**) The z-pin/matrix interface crack initiated (**b**) the z-pin/matrix interface was partially debonded and delamination initiated (**c**) z-pin and matrix were deformed (**d**) the z-pin ruptured and the specimen failed.

In the present study, interlaminar strengthening mechanism of z-pinning in the delamination of metal matrix composites is determined by an in situ double-notched interlaminar shear test. Figure 9 shows the schematic of failure process of the z-pinned composites. The failure process of a z-pinned composite subjected to interlaminar shear load is defined in four stages. Initially, micro-cracks are observed in the z-pin/matrix interface due to stress concentration, which has a negligible effect on load transfer through the interface. Thus, the z-pin can carry the main load because of the strong interface bonding and the high strength of the z-pin itself, which improves the interlaminar shear strength of the z-pinned Cf/Al specimen, as shown in Figure 9a. Secondly, the z-pin/matrix interface is partially debonded due to the gradual development of the interface crack. Thus, delamination initiates near the notches and the load drops sharply until its value is close to the interlaminar shear strength of the unpinned Cf/Al composites, as shown in Figure 9b. Then, with the increase in displacement, the z-pin/matrix interface is fully debonded, and the interlaminar cracks extend to the vicinity of the z-pin. Following the interface debonding, the z-pin experiences a small amount of frictional slide along the interface and an irreversible shear deformation. The aluminum matrix is deformed and ploughed out due to lateral deflection of the z-pin, which results in an enhanced frictional region near the delamination fracture surface. The enhanced frictional region partially contributes to the interlaminar shear strength of this stage, as shown in Figure 9c. Finally, the interlaminar shear stress causes the failure of the z-pin by rupture, which eventually leads to the failure of the specimen, as shown in Figure 9d.

## 4. Conclusions

In this study, we carried out an in situ double-notched interlaminar shear test to investigate the delamination suppression mechanisms of the z-pin in carbon fiber-reinforced aluminum alloy composites (Cf/Al) composites. The experimental results determined that z-pinning is highly effective in enhancing the delamination resistance of Cf/Al composites. Introducing the stainless-steel z-pin improved the interlaminar shear strength of Cf/Al composite by 148%. The interlaminar strengthening effect depends on the interface bonding situation. Initially, the z-pinned specimen showed an improved interlaminar strength due to the strong interfacial bonding and the z-pin’s high strength. When the interface was debonded, the enhanced frictional region exerted by the deformed z-pin contributed to the interlaminar shear strength of the specimen. The failure process of the z-pinned composite is complex and consisted of carbon fiber/aluminum interfacial debonding, z-pin/aluminum interfacial debonding, aluminum plough, z-pin shear deformation, z-pin frictional sliding, z-pin rupture, and composite delamination.

## Figures and Tables

**Table 1:** Basic properties of carbon fibers, z-pin and 5A06 Al alloy.

Materials	Tensile Strength (MPa)	Elastic Modulus (GPa)	Elongation to Fracture (%)	Density (g/cm^3^)
M40	4410	377.0	1.2	1.76
AISI 321	1905	198.0	2.0	7.85
5A06 Al	314	66.7	16.0	2.64

**Table 2:** Chemical composition of 5A06 Al alloy (wt%).

Material	Mg	Mn	Si	Fe	Zn	Cu	Ti	Al
5A06 Al	5.8–6.8	0.5–0.8	0.4	0.4	0.2	0.1	0.02–0.1	Bal.

**Table 3:** Chemical composition of AISI 321 (wt%).

Material	Cr	Ni	Ti	Mn	Si	C	S	P	Fe
AISI 321	17–19	8–11	0.5–0.8	<2.0	<1.0	<0.12	<0.03	<0.035	Bal.

**Table 4:** Interlaminar shear strengths of controls and z-pinned specimens.

Specimen	Interlaminar Shear Strength	Standard Deviation
control specimen	13.6 MPa	0.78
z-pinned specimen with half z-pin	33.7 MPa *	1.94
z-pinned specimen with entire z-pin [10]	38.4 MPa	1.96

*: The experimental values were 31.2, 34.1, and 38.4 MPa, respectively. The value of 34.1 MPa is discussed in detail in the present research.

## References

[1] Li D.G., Chen G.Q., Jiang L.T., Xiu Z.Y., Zhang Y.H., Wu G.H. (2013). Effect of thermal cycling on the mechanical properties of Cf/Al composites. Mater. Sci. Eng. A.

[2] Pei R., Chen G., Wang Y., Zhao M., Wu G. (2018). Effect of interfacial microstructure on the thermal-mechanical properties of mesophase pitch-based carbon fiber reinforced aluminum composites. J. Alloy. Compd..

[3] Zhang Y.H., Wu G.H. (2006). Interface and thermal expansion of carbon fiber reinforced aluminum matrix composites. Trans. Nonferrous Met. Soc. China.

[4] Li M., Matsuyama R., Sakai M. (1999). Interlaminar shear strength of C/C-composites: The dependence on test methods. Carbon.

[5] Pingkarawat K., Wang C.H., Varley R.J., Mouritz A.P. (2012). Self-healing of delamination cracks in mendable epoxy matrix laminates using poly[ethylene-co-(methacrylic acid)] thermoplastic. Compos. Part A.

[6] Mouritz A.P. (2007). Review of z-pinned composite laminates. Compos. Part A.

[7] Wang S., Wang Y.H., Wu G.H. (2018). Interlaminar shear properties of z-pinned carbon fiber reinforced aluminum matrix composites by short-beam shear test. Materials.

[8] Pegorin F., Pingkarawat K., Daynes S., Mouritz A.P. (2014). Mode II interlaminar fatigue properties of z-pinned carbon fibre reinforced epoxy composites. Compos. Part A.

[9] Koh T.M., Isa M.D., Feih S., Mouritz A.P. (2013). Experimental assessment of the damage tolerance of z-pinned T-stiffened composite panels. Compos. Part B.

[10] Zhang Y.H., Yan L.L., Miao M.H., Wang Q.W., Wu G.H. (2015). Microstructure and mechanical properties of z-pinned carbon fiber reinforced aluminum alloy composites. Mater. Des..

[11] Knaupp M., Baudach F., Franck J., Scharr G. (2014). Mode I and pull-out tests of composite laminates reinforced with rectangular z-pins. J. Compos. Mater..

[12] Cartié D.D.R., Cox B.N., Fleck N.A. (2004). Mechanisms of crack bridging by composite and metallic rods. Compos. Part A.

[13] Naseem K., Yang Y., Luo X., Huang B., Feng G. (2011). SEM in situ study on the mechanical behaviour of Si Cf/Ti composite subjected to axial tensile load. Mater. Sci. Eng. A.

[14] Boesl B., Lahiri D., Behdad S., Agarwal A. (2014). Direct observation of carbon nanotube induced strengthening in aluminum composite via in situ tensile tests. Carbon.

[15] Su J., Wu G.H., Li Y., Gou H.S., Chen G.H., Xiu Z.Y. (2011). Effects of anomalies on fracture processes of graphite fiber reinforced aluminum composite. Mater. Des..

[16] Zhang B., Allegri G., Yasaee M., Hallett S.R. (2015). Micro-mechanical finite element analysis of Z-pins under mixed-mode loading. Compos. Part A.

[17] Yasaee M., Lander J.K., Allegri G., Hallett S.R. (2014). Experimental characterisation of mixed mode traction-displacement relationships for a single carbon composite Z-pin. Compos. Sci. Technol..

[18] Cox B.N. (2005). Snubbing effects in the pullout of a fibrous rod from a laminate. Mech. Adv. Mater. Struct..

[19] Cui H., Li Y., Koussios S., Zu L., Beukers A. (2011). Bridging micromechanisms of Z-pin in mixed mode delamination. Compos. Struct..

[20] Zhang Y., Wu G. (2010). Comparative study on the interface and mechanical properties of T700/Al and M40/Al composites. Rare Met..

